# Inclisiran: A Systematic Review Exploring the Revolutionary Approach of Twice-Yearly Dosing Regimen in the Treatment of Hypercholesterolemia

**DOI:** 10.7759/cureus.69918

**Published:** 2024-09-22

**Authors:** Sanjana Singareddy, Surakchhya Dhakal, Therese Anne Limbaña, Vignesh Murugan, Farhana Nazmin, Jian Garcia, Safeera Khan

**Affiliations:** 1 Internal Medicine, California Institute of Behavioral Neurosciences & Psychology, Fairfield, USA

**Keywords:** hyperlipidemia treatment, inclisaran, lipid lowering agents, rna interference technology, small interfering rna

## Abstract

The global burden of hyperlipidemia is on the rise, along with an increase in associated cardiovascular complications. Since most of the patients affected by hyperlipidemia are elderly individuals with multiple comorbidities, the introduction of even a single additional drug for asymptomatic conditions such as hyperlipidemia can drastically reduce treatment compliance due to their long medication history. Hence, researchers are trying to come up with a drug with a long duration of action requiring less frequent dosing without compromising compliance and improving the outcome. This led to the discovery of inclisiran, a "wonder drug" that utilizes small interference RNA and requires only twice-yearly administration to maintain patients' lipid levels at optimal levels. We conducted a systematic review by following standardized guidelines on the long-term efficacy and safety of the new drug, inclisiran, in the treatment of hypercholesterolemia.

We conducted an advanced search on PubMed using the MeSH strategy and then employed appropriate keywords to search other major databases, such as PubMed Central and Medline, using various inclusion and exclusion criteria, which yielded 94 articles from various databases. We narrowed down the search to 10 randomized controlled trials (ORION trials) after removing duplicates and screening for irrelevant titles for inclusion in the study. The ORION trials on inclisiran evaluated the drug's impact on various parameters, such as low-density lipoprotein-cholesterol (LDL-C), proprotein convertase subtilisin/kexin type 9 (PCSK9), high-density lipoprotein (non-HDL), apolipoprotein B (apoB), and so on, while considering the safety aspects of the drug. All the trials indicate greater efficacy of inclisiran and long-term maintenance of the results achieved when compared to a placebo and showed a long dosing interval, thereby increasing treatment compliance.

Additionally, as the drug's dose increased, we observed greater reductions in the mentioned parameters without a significant increase in the incidence of adverse events. According to the review's data analysis, inclisiran, with its greater efficacy, has the potential to replace conventional pharmacological therapy in the near future, with the best results achieved when combined with lifestyle modifications. However, a long-term assessment of the drug's efficacy and safety is required before implementing it in clinical practice to identify any potential safety concerns, particularly related to the administration of higher dosage over a longer period.

## Introduction and background

As stated by Dr. Christopher Cannon, "Hyperlipidemia is not a disease, but a pathologic condition that can contribute to the development of cardiovascular diseases." This condition often goes undiagnosed as it is symptom-free and hence untreated, leading to the increased prevalence of the disease, and contributing to a continuous rise in mortality and morbidity due to associated cardiovascular complications. According to reports published in the Journal of the American College of Cardiology, only 55% of the adult population in the United States were aware of their condition, and only a few received treatment [1]. By 2017, approximately 39% of adults were affected by hyperlipidemia worldwide, and by 2021, about 29.8% of the US population aged 20 years and older were living with hyperlipidemia [2,3]. Per the lobal statistics presented by the American Heart Association, around 23.7% of cardiovascular deaths were attributed to dyslipidemia globally, indicating the significant role of elevated levels of low-density lipoprotein-cholesterol (LDL-C) in cardiovascular mortality [4].

Hyperlipidemia can be defined as higher than normal levels of either one or more lipids in the plasma, which is classified clinically as hypercholesterolemia, hypertriglyceridemia, or mixed hyperlipidemia depending on the elevated lipid [5]. When the level of total cholesterol exceeds 240 mg/dl (6.2 mmol/L) or if the triglyceride level exceeds 200 mg/dl (2.26 mmol/L), the risk of atherosclerotic events such as coronary artery disease, cerebral infarction, peripheral vascular disease is increased significantly [5-7]. Among the lipids, LDL-C is identified as the major contributor to atherosclerotic events and is currently the primary target for lipid-lowering therapy [8,9].

The conventional approach to treat hyperlipidemia includes the use of statins that target cholesterol synthesis in the liver by inhibiting the enzyme HMG-CoA reductase, combined with dietary regulations, exercise therapy, and lifestyle modifications [10]. However, the recent trend leans towards the use of personalized approach with monoclonal antibodies targeting proprotein convertase subtilisin/kexin type 9 (PCSK9), such as evolocumab; utilization of RNA interference technology (inclisiran); genetic editing techniques like CRISPR-cas9; identifying novel lipid targets beyond LDL such as lipoprotein(a), apolipoprotein B (apoB), and triglyceride-rich lipoproteins [11].

The current systematic review focuses on the one drug that has gained significant attention in the field of research due to its unique requirement of dosing just twice a year, which could have the potential to dramatically improve compliance without compromising on the efficacy of the lipid-lowering, thereby significantly reducing the burden of life-threatening comorbidities associated with hyperlipidemia. This review also focuses on the practical implementation of inclisiran in regular practice by assessing considerations related to its safety in comparison to placebo.

## Review

Methods 

Search Strategy

By strictly adhering to the guidelines of Preferred Reporting Items for Systematic Reviews and Meta-analysis (PRISMA), a comprehensive search of various electronic databases was performed to gather relevant literature on inclisiran and its role in the management of hyperlipidemia. Using appropriate regular and Medical Subject Headings (MeSH) keywords, the major databases PubMed, PubMed Central, and Medline were searched to obtain the required articles. The search algorithm-related limitations in PubMed advanced search were mitigated by using appropriate keywords in PubMed Central and Medline. To limit data overload, only the above-mentioned databases were included in the search. The keywords used in the literature search included ‘Inclisiran’, ‘RNA interference technology’, ‘small interfering RNA’, ‘hyperlipidemia’, and ‘lipid-lowering agents’.

The final MeSH strategy used in the PubMed was as follows - ("RNA, Small Interfering/administration and dosage"[MeSH] OR "RNA, Small Interfering/adverse effects"[MeSH] OR "RNA, Small Interfering/classification"[MeSH] OR "RNA, Small Interfering/drug effects"[MeSH] OR "RNA, Small Interfering/metabolism"[MeSH] OR "RNA, Small Interfering/therapeutic use"[MeSH] OR "RNA, Small Interfering/toxicity"[MeSH]) AND ("Hyperlipidemias/drug therapy"[MeSH] OR "Hyperlipidemias/prevention and control"[MeSH] OR "Hyperlipidemias/therapy"[MeSH]) - as presented in Table 1.

**Table 1:** Search strategy MeSH: Medical Subject Headings; RNA: ribonucleic acid

Database	Search strategy
PubMed, PubMed Central	("RNA, Small Interfering/administration and dosage"[MeSH] OR "RNA, Small Interfering/adverse effects"[MeSH] OR "RNA, Small Interfering/classification"[MeSH] OR "RNA, Small Interfering/drug effects"[MeSH] OR "RNA, Small Interfering/metabolism"[MeSH] OR "RNA, Small Interfering/therapeutic use"[MeSH] OR "RNA, Small Interfering/toxicity"[MeSH]) AND ("Hyperlipidemias/drug therapy"[MeSH] OR "Hyperlipidemias/prevention and control"[MeSH] OR "Hyperlipidemias/therapy"[MeSH])
Medline	Keywords - ‘Inclisiran’, ‘RNA interference technology’, ‘small interfering RNA’, ‘hyperlipidemia’, ‘lipid-lowering agents’

The relevant articles obtained through PubMed advance search using the MeSH strategy, and other databases, were transferred to EndNote Basic. The search results were assessed independently by two reviewers. The articles from EndNote were exported to Microsoft Excel and duplicates were removed. After deleting duplicates, titles were screened for irrelevant articles and those found were removed. The remaining articles were subjected to full-text and abstract screening, and articles that did not focus on the topic of interest were discarded. The selected articles were then evaluated by quality assessment using the Jadad scale. Only high-quality articles were considered for inclusion in the systematic review.

Inclusion and Exclusion Criteria

The articles had to be in the English language to be included in the study. Only original articles, and observational studies published after 2014 were taken into consideration. Systematic reviews, meta-analyses, traditional reviews, editorials, perspectives, case reports, peer reviews, unpublished studies, and animal studies were excluded. The inclusion and exclusion criteria are summarized in Table 2.

**Table 2:** Inclusion and exclusion criteria RCT: randomized controlled trial

Characteristics	Inclusion criteria	Exclusion criteria
Language	Literature published in the English language	Literature published in languages other than English
Type of study	Observational studies, RCTs	Articles other than observational studies or RCTs
Year of publishing	Articles published later than 2014	Articles published before 2014
Content of the study	Articles with content aligning parallel to the research question	Articles with content not applicable to the research question of interest
Disease status of study participants	Participants must be diagnosed with hyperlipidemia before the study design	Studies involving participants without hyperlipidemia

Analysis of the Quality of the Study/Bias

Twenty-two articles for finalized for quality assessment after removing duplicates, excluding irrelevant titles, and abstract and full text-screening of the articles obtained via above mentioned electronic databases. Among the 22 articles, 10 were categorized as high- or medium-quality papers and were included in the study [12-21]. The quality assessment tool utilized in the analysis of articles was the Jadad scale for randomized controlled trials (RCTs). The quality assessment report of the included studies is presented in Table 3.

**Table 3:** Quality assessment of the included studies by the Jadad scale

Jadad scale	Ray et al., 2017 [12]	Ray et al., 2019 [13]	Ray et al., 2023 [14]	Ray et al., 2020 [15]	Raal et al., 2018 [16]	Raal et al., 2020 [17]	Wright et al., 2024 [18]	Luo et al., 2023 [19]	Yamashita et al., 2024 [20]	Huo et al., 2024 [21]
1. Was the method of randomization adequate?	Yes	Yes	Yes	Yes	Yes	Yes	Yes	Yes	Yes	Yes
2. Was the treatment allocation concealed?	Yes	Yes	No	Yes	Yes	Yes	Yes	Yes	Yes	Yes
3. Were study participants and providers blinded to treatment group assignment?	Yes	Yes	No	Yes	Yes	Yes	Yes	Yes	Yes	Yes
4. Were the people assessing the outcomes blinded to the participants' group assignments?	No	No	No	No	No	No	No	No	No	No
5. Were the groups similar at baseline on important characteristics that could affect outcomes?	Yes	Yes	Yes	Yes	Yes	Yes	Yes	Yes	Yes	Yes
6. Was the overall drop-out rate from the study at endpoint 20% or lower of the number allocated to treatment?	Yes	Yes	Yes	Yes	Yes	Yes	Yes	Yes	Yes	Yes
7. Was the differential drop-out rate at endpoint 15 percentage points or lower?	Yes	Yes	Yes	Yes	Yes	Yes	Yes	Yes	Yes	Yes
8. Was there high adherence to the intervention protocols for each treatment group?	Yes	Yes	Yes	Yes	Yes	Yes	Yes	Yes	Yes	Yes
9. Were other interventions avoided or similar in the groups?	Yes	Yes	Yes	Yes	Yes	Yes	Yes	Yes	Yes	Yes
10. Were outcomes assessed using valid and reliable measures, implemented consistently across all study participants?	Yes	Yes	Yes	Yes	Yes	Yes	Yes	Yes	Yes	Yes
11. Did the authors report that the sample size was sufficiently large to be able to detect a difference in the main outcome between groups with at least 80% power?	Yes	Yes	Yes	Yes	Yes	Yes	Yes	No	Yes	Yes
12. Were all randomized participants analyzed in the group to which they were originally assigned, i.e., did they use an intention-to-treat analysis?	Yes	Yes	Yes	Yes	Yes	Yes	Yes	Yes	Yes	Yes
Overall score	11/12	11/12	9/12	11/12	11/12	11/12	11/12	10/12	11/12	11/12

Results

**Figure 1:**
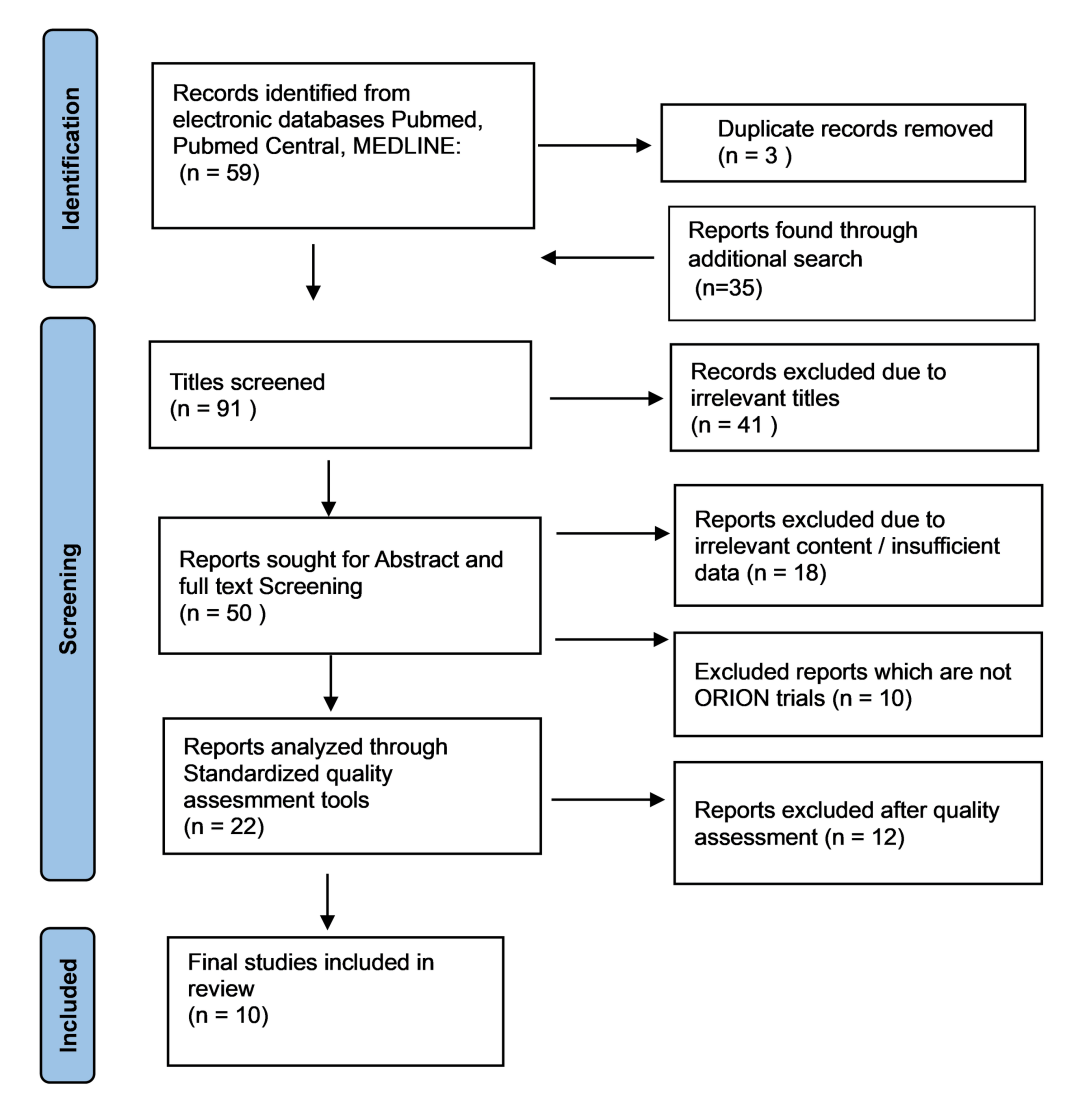
PRISMA flow chart depicting the selection of studies PRISMA: Preferred Reporting Items for Systematic Reviews and Meta-Analysis

We obtained 94 papers from the online database using the MeSH strategy and relevant keywords as part of our search strategy. Following the deletion of duplicates, we subjected 91 papers to title screening. After removing articles with irrelevant titles, we selected 50 articles for abstract and full-text screening. Two personnel carried out abstract and full-text screening to exclude articles with content irrelevant to the topic of interest and narrowed it down to 22 articles. A PRISMA flow chart of the search results is given in the figure below (Figure 1) [22]. To maintain uniformity and evaluate the impact of inclisiran on test subjects and the obtained results, we only selected the ORION trials for inclusion in the study before subjecting the articles to quality checks. Only 12 of the 22 selected articles underwent quality assessment, and the current study included 10 high-quality ORION trials.

The included studies consist of trials: ORION-1, one-year follow-up of ORION-1, four-year follow-up of ORION-1 (ORION-3), ORION-5, ORION-7, ORION-9, ORION-10 and ORION-11, ORION-14, ORION-15, ORION-18 and the characteristics of included studies were given in the table below (Table 4). The results of the aforementioned trials consistently indicated that inclisiran is more effective and maintains results over a longer period compared to Placebo. Additionally, its extended dosing interval contributes to increased treatment compliance. And, as the drug's dose increases, we have observed higher reductions in the previously mentioned parameters, without any significant increase in the incidence of adverse events (p<0.001). Also, the adverse events were comparable to those of placebo, with similar incidence rates. All the trials reported similar outcomes in reducing the parameters mentioned, and the results were in favor of the possible implementation of inclisiran in clinical practice in the near future.

**Table 4:** Characteristics of the included studies CI: confidence interval; ASCVD: atherosclerotic cardiovascular disease; HeFH: heterozygous familial hypercholesterolemia; HoFH: homozygous familial hypercholesterolemia; LDL-C: low-density lipoprotein cholesterol; PCSK 9: proprotein convertase subtilisin/kexin type 9

Study	ORION trial	Aim of the study	Study population	Results
Ray et al., 2017 [12]	ORION-1	To evaluate the efficacy of the drug inclisiran in achieving the primary outcome with increasing doses of the drug when compared to placebo	High cardiovascular risk patients with elevated LDL levels (n=497)	Inclisiran lowered the levels of LDL and PCSK 9 effectively, with higher doses achieving greater reductions without any increase in the rate of adverse events
Ray et al., 2019 [13]	One-year follow-up study of ORION-1	To evaluate the efficacy of inclisiran in achieving mean reductions in LDL with an infrequent dosing regimen and the sustainment of results achieved	Followed 497 patients from the ORION-1 trial for one year	Both one and two doses of inclisiran regimens offered an average reduction in LDL by 29.5% to 46.4% with a 50% reduction in LDL-C maintained over six months after two doses offering a potent, sustainable lipid-lowering therapeutic option with infrequent dosing
Ray et al., 2023 [14]	ORION-3	To evaluate the long-term efficacy of inclisiran in achieving the primary outcome of reducing LDL-C and PCSK9 levels with a continued twice-yearly treatment regimen in high-cardiovascular-risk patients and elevated LDL cholesterol levels	Four-year follow-up of 497 patients from the ORION-1 trial	With twice-yearly dosing, inclisiran was able to achieve satisfying reductions in the levels of LDL-C (4-year averaged mean reduction of 44·2% (95% CI: 47·1–41·4) and PCSK9 (4-year averaged mean reduction of −69·5% (95% CI: −71·2 to −67·9), and the reductions have sustained over 1440 days
Ray et al., 2020 [15]	ORION – 10 and 11	To evaluate the efficacy of the drug inclisiran in achieving corrected and time-adjusted percentage changes in levels of LDL-C from baseline	Two groups of 1561 patients with atherosclerotic cardiovascular disease and LDL-C above 70mg/dl, 1671 patients with atherosclerotic cardiovascular risk factors and LDL-C above 100mg/dl constituting ORION-10, ORION-11 trials respectively	Inclisiran was able to achieve a reduction of approximately 50% in the levels of LDL-C (by 52.3% (95% confidence interval [CI], 48.8 to 55.7) in the ORION-10 trial and by 49.9% (95% CI: 46.6 to 53.1) in the ORION-11 trial) when administered subcutaneously every six months
Raal et al., 2018 [16]	ORION-5	To evaluate the efficacy, safety, and tolerability of inclisiran in patients with HoFH and elevated LDL-C levels despite maximum tolerated doses of LDL-C–lowering therapies	Phase I of the study consists of 56 patients with HoFH, and 53 out of 56 patients continued an 18-month open-label phase II	Inclisiran treatment did not reduce LDL-C levels (placebo-corrected percentage change in LDL-C level from baseline to day 150 was −1.68% (95% CI: −29.19% to 25.83%; P=0.90) in patients with homozygous familial hypercholesterolemia despite substantial lowering of PCSK9 levels. (placebo-corrected percentage change in PCSK9 levels from baseline to day 150 was −60.6%, p<0.0001)
Raal et al., 2020 [17]	ORION-9	To evaluate the efficacy, safety, and tolerability of inclisiran in patients with HeFH and elevated LDL-C levels	Patients with HeFH with elevated LDL levels above 100mg/dl (n=482)	Inclisiran significantly lowered levels of LDL cholesterol (percent reduction of 39.7% as compared to 8.2% with placebo) than those who received placebo, with an infrequent dosing regimen and an acceptable safety profile
Wright et al., 2024 [18]	ORION-8	To evaluate the long-term efficacy and tolerability of inclisiran in adults through the addition of a twice-yearly dosage of inclisiran in an open-label extension trial of ORION-9, ORION-10, ORION-11, and ORION-3	Adult patients with atherosclerotic cardiovascular disease (ASCVD), ASCVD risk equivalent, or HeFH (n=3275)	In the largest and longest follow-up to date with >12 000 patient-years exposure, inclisiran demonstrated consistent and effective LDL-C lowering [78.4% of patients achieved pre-specified LDL-C goals and mean percentage change in LDL-C was −49.4% (95% CI: −50.4, −48.3)] with a favorable long-term safety and tolerability profile.
Luo et al., 2023 [19]	ORION-14	To evaluate the safety, pharmacokinetics, and LDL-C lowering effects of inclisiran in Chinese patients with elevated LDL-C despite treatment with maximally tolerated LDL-C lowering therapies.	Chinese patients with hypercholesterolemia (LDL-C >100mg/dl) who were on maximally tolerated statin (n=40)	A single dose of both inclisiran 100 and 300 mg significantly reduced PCSK9 and LDL-C levels (56.4% and 49.6% of 100 mg, 74.9% and 58.3% of 300 mg, respectively) with the greatest reductions observed in 300 mg inclisiran arm
Yamashita et al., 2024 [20]	ORION-15	To evaluate the efficacy, safety, and pharmacokinetics of inclisiran in Japanese patients with high cardiovascular risk and elevated LDL-C levels.	Japanese patients with hypercholesterolemia, including HeFH (n=312)	Inclisiran sodium 100, 200, and 300 mg demonstrated statistically significant LDL-C and PCSK9 reductions at Day 180 (The greatest reductions of LDL-C, 65.3%; PCSK9, 79.2% were with inclisiran sodium 300 mg with more than 86% of the patients receiving inclisiran achieving the Japan Atherosclerosis Society 2017 lipid management targets), which were consistent over 12 months
Huo et al., 2023 [21]	ORION-18	To evaluate the efficacy and safety of inclisiran in Asian patients with ASCVD or high risk of ASCVD, as an adjunct to diet and maximally tolerated statin dose	Patients with atherosclerotic cardiovascular disease (ASCVD) or high risk of ASCVD (n=345)	Inclisiran was effective and safe in Asian Participants With ASCVD or ASCVD High Risk and Elevated LDL-C [percentage decrease in LDL-C, PCSK 9 levels from baseline to day 330 were 57.2%, 78.3% respectively (p<0.001 for both)]

Discussion

Inclisiran (trade name Leqvio®) is a novel lipid-lowering agent approved for the treatment of hypercholesterolemia, dyslipidemia, and familial hypercholesterolemia [23]. It is a short-chain Small interfering RNA (siRNA) molecule that binds to GalNac and the asialoglycoprotein receptor (ASGPR) on the membranes of liver cells, where inclisiran binds to the RNA-induced silencing complex (RISC) which cleaves the target mRNA. It also binds to PCSK9-encoding mRNA, leading to decreased levels of PCSK9 promoting lysosomal degradation of LDL receptors, thereby lowering LDL-C levels [24]. Inclisiran exhibits a prolonged lipid-lowering effect, a favorable safety profile, and various advantages over conventional therapies. 

A single dose of inclisiran has the potential to lower LDL-c levels by about 50% and maintain the reduction for nearly six months of duration and the silencing complex was also found to be active even after the degradation of mRNA requiring just twice a year dosing to maintain the results, thereby improving adherence [25,26]. Twice-a-year dosing was found to be sufficient to maintain a significant lowering of LDL-C levels, thus improving adherence, and is now considered an attractive option in overcoming non-adherence, thereby possibly improving cardiovascular mortality and morbidity. The first ORION trial of inclisiran was carried out by Ray et al. in 2017, and over the past few years, many phase III randomized double-controlled trials (ORION) have been conducted to evaluate the safety, efficacy, and impact on various atherogenic lipoproteins. The current systematic review includes summaries of the ORION trials, along with a detailed assessment of the impact of inclisiran therapy on various parameters.

Impact of Inclisiran on LDL-C Reduction

Ray et al. conducted the first trial of ORION-1 in 2017 to evaluate the effect of various doses of inclisiran and the frequency of dosing intervals on lipid levels in 501 patients whose LDL-C levels were above 70 mmHg with a history of atherosclerotic disease or above 100 mmHg in those without any atherosclerotic risk factors. Inclisiran was administered in various doses and frequencies according to the randomization along with two controlled placebo groups (200 mg/300 mg/500 mg single dose of inclisiran or 100 mg/200 mg/300 mg of inclisiran on days 1 and 90 comprising 60 patients in each group). All the patients were followed up to either day 210 or until the LDL levels were reduced by 20%. At day 180, percentage changes in LDL-C levels were calculated from baseline and, a single dose of inclisiran achieved a mean reduction of 27.9% to 41.9% as compared to placebo (2.1% increase), with the reductions ranging between 28.2% and 41.9% by the end of day 240.

The addition of a second dose of inclisiran resulted in a significant reduction in LDL-C levels, which decreased by 35.5% to 52.6% at day 180 and by 26.7% to 47.2% at day 240. The study also noted reductions in absolute levels of LDL-C, with a mean of -64.2 +20.7 mg/dl at day 18, and 66% of patients achieved levels of 70 mg/dl with 300 mg of inclisiran, 48% achieved levels of 50 mg/dl, and 5% achieved levels of 25 mg/dl. The reduction in LDL levels was found to be maintained by the end of day 240, thus sustaining a long-term reduction with just one or two doses of inclisiran (p=0.001 for all comparisons vs. placebo) [12].

The patients of the ORION-1 trial were followed up for a year after the end of the study period without administering additional doses of inclisiran. At day 360, the single-dose group saw reductions in LDL-C levels ranging from 15.4% to 21.6%, while the 2-dose group saw decreases ranging from 12.6% to 31.4%. The 2-dose 300 mg inclisiran regimen showed the maximum effects, suggesting greater reductions with a higher dosage and increased frequency. The gain in time-averaged LDL-C reduction over one year with 500 mg vs. 300 mg single dose was modest at 2.1%. However, the administration of a second 300-mg dose at day 90 resulted in an additional 10% reduction in LDL-C levels. It was concluded that by the end of day 330, 60-75% of patients who received one dose of inclisiran and 69.5-86.3% of those receiving two doses had not returned to within a 20% change in baseline LDL-C levels, and the duration of return to baseline levels was found to be dose-dependent, with most participants not returning to 20% of baseline levels. The patients maintained an LDL-C reduction of at least 39 mg/dl for a median of six to seven months from baseline with a single dose and 5-10 months with two doses, suggesting sustained lipid-lowering therapy with inclisiran [13].

A four-year open-label extension of the ORION-1 trial, called ORION-3, was carried out, to study the percentage changes in LDL cholesterol levels from baseline. The subjects in the inclisiran arm of ORION-1 received 300 mg subcutaneous inclisiran twice a year, while those on placebo in ORION-1 received 140 mg subcutaneous evolocumab every two weeks for up to one year before switching to 300 mg subcutaneous inclisiran sodium providing around a huge 1209.6 patient-years of cumulative exposure to inclisiran beginning from the ORION-1 in the treatment arm and 250.2 patient-years in switching arm. In the inclisiran arm, LDL-C levels at day 210 showed a reduction of 47.5% (95% confidence interval of -50.7 to -44.3, P 0.001). The mean percentage reduction in LDL was estimated to be between 34.3% and 53.8%, with an absolute reduction of 1.13 mmol/L to 1.76 mmol/L, with just two injections of inclisiran per year providing an overall four-year time-averaged reduction of 44.2% [14].

The researchers conducted two more phase-3 double-blind randomized control trials that were similar to ORION-1. These were ORION-1O (1561 participants with atherosclerotic cardiovascular disease and LDL levels above 70 mg/dl) and ORION-11 (1617 atherosclerotic cardiovascular disease risk equivalent patients with LDL levels above 100 mg/dl), which added up to 2166 person-years of exposure to inclisiran. The patients were randomly assigned either 284 mg inclisiran (equivalent to 300 mg inclisiran sodium) or placebo on days 1, 90, and every six months thereafter over 540 days. The people with atherosclerosis had 51.3% lower LDL levels at day 510, while the placebo group only had 1.0% lower levels with a between-group difference of -52.3% (95% CI: 55.7 to 48.8; p<0.001).

The time-adjusted percent reduction in LDL levels was also -51.3% as compared to a 2.5% reduction with a placebo. However, a slightly lower rate of reduction was noted in the individuals with atherosclerotic risk equivalents as compared to the atherosclerotic CV disease subjects. The percentage reduction and time-adjusted percent reduction were both found to be -45.8% in the inclisiran group as compared to 4.05 and 3.4% with placebo, respectively. The absolute reduction in LDL levels, as well as the time-adjusted absolute change in LDL levels, are -56.2 mg/dl, -53.7 mg/dl in the ORION-10 trial, and -50.9 mg/dl, -48.7 mg/dl in the ORION-11 trial, respectively, following the trend of lower rates of reduction than in the counter group patients of the ORION-10 trial [15].

After the successful reduction of LDL levels by Inclisiran in the above-mentioned initial ORION trials, Ray et al. have conducted two phase 3 randomized trials across various countries to evaluate the efficacy, safety, and tolerability of inclisiran in patients with homozygous familial hypercholesterolemia (HoFH) and heterozygous familial hypercholesterolemia (HeFH) named ORION-5 and ORION-9, respectively. ORION-5 was conducted in two phases. Part one consisted of a six-month double-blinded placebo-controlled phase with 56 patients randomized to either 300 mg subcutaneous inclisiran or placebo on days 1 and 90. The second phase consisted of an 18-month open-label phase where the subjects in the placebo group were transitioned to inclisiran on day 180, with all the phase 2 subjects (n=53) receiving subsequent doses of inclisiran therapy till day 720. The final results revealed that inclisiran has lowered the placebo-corrected percentage in LDL levels from baseline to 1.68%, and the absolute change was -6.47 mg/dl in phase one with no statistical or clinical significance [16].

In ORION-9, study subjects received subcutaneous injections of 300 mg inclisiran sodium or matching placebo on days 1, 90, 270, and 450. At day 510, percentage changes in LDL-C levels from baseline were calculated and found that the levels reduced by 39.7% in the inclisiran group as compared to increased LDL-C levels by 8.2% in the placebo group, with a huge between-group difference of -47.9%. The time-adjusted percent change from baseline in LDL levels between days 90 and 540 was reduced by 38.1% in the inclisiran group and increased by 6.2% in the placebo group, with a between-group reduction of 44.3% and robust reductions observed in all genotypes of HeFH. There was also a decrease of 59.0 mg/dl in absolute levels of LDL in the inclisiran group with an increase of 9.9 mg/dl in the placebo group, with a time-adjusted reduction of -56.9 mg/dl and -5.8 mg/dl between days 90 and 540 in the inclisiran and placebo groups, respectively. However, the reductions observed in the homozygous group of the ORION-5 trial were not found to be significant, unlike the reductions found in the ORION-9 trial, and further studies might be needed to evaluate the effects of inclisiran in HoFH patients [17].

A multicenter long-term extension trial of the above trials ORION-3, ORION-9, ORION-10, and ORION-11 was conducted across 268 centers in 13 countries with nearly 80% of the combined parent trial populations named ORION-8, where the date of the end-of-study visit for the parent trial was considered day 1 of ORION-8. Subjects who received blinded inclisiran in parent trials ORION 9-11 were given a blinded placebo on day 1, and those who received blinded placebo in the above parent trials received blinded inclisiran sodium. Meanwhile, subjects who were rolled over from the ORION-3 trial did not receive any study medication on day 1. At day 90, all the subjects were transitioned to open-label 300 mg inclisiran sodium, and the subjects from ORION 9-11 were followed until day 1080 or until >90 days after the last dose of inclisiran. However, subjects from ORION-3 were only followed for a shorter duration of 1.7 years (ORION-3 is itself a four-year follow-up study of ORION-1).

This trial provides additional exposure to inclisiran, corresponding to a mean of 2.6 years and 8530 patient-years of exposure in ORION-8, and when combined with parent trials, it offered a mean exposure of 3.7 years, with 12,109 patient-years of exposure with a maximum mean of 6.8 years, where over a quarter of patients were exposed for over 4.5 years. By the end of the study, a total of 79.4% achieved the prespecified goal of 70 mmHg in the ASCVD group, and a total of 74.3% achieved the goal of <100 mmHg in ASCVD risk-equivalent groups. In the overall population, the mean percentage change in LDL was -49.4% and the mean absolute change was -1.5 mmol/L by the end of the study, suggesting benefit from long-term inclisiran exposure [18].

Similar reductions in LDL-C levels were noted in other trials like ORION-14 and ORION-15, where the former was conducted in a group of Chinese subjects with hypercholesterolemia (LDL levels >100 mg/dl) to evaluate the pharmacological properties along with safety following a single dose of subcutaneous 100 mg and 300 mg inclisiran, which revealed a greater reduction in LDL with an earlier onset of action with the 300 mg dose, and the maximum reduction of 63.9% was observed on Day 30. The lipid-lowering effect of single-dose inclisiran sustained till the end of the study date, day 90, with 49.6% and 58.3% reductions in the 100 mg and 300 mg groups, respectively, as compared to 19.3% reduction with placebo, ensuring long-term maintenance on a single-dose regimen [19].

The latter trial, ORION-15, was similar to the former but carried out on Japanese subjects by Yamashita et al. in a study designed to assess the efficacy, safety, and pharmacokinetics (PK) of the drug in patients with high cardiovascular risk factors and elevated LDL-C levels, including those with heterozygous familial hypercholesterolemia (HeFH). On days 1, 90, and 270, Yamashita et al. randomly assigned the study subjects to receive either 100 mg, 200 mg, 300 mg subcutaneous inclisiran sodium or placebo subcutaneously. At day 180, the percentage changes in LDL-C from baseline were -56.6%, -60.9%, and -65.3% for 100 mg, 200 mg, and 300 mg doses, respectively, revealing a greater reduction with higher doses. The placebo-adjusted absolute changes from baseline were -62.5 mg/dl, -67.1 mg/dl, and -70.9 mg/dl for doses 100 mg, 200 mg, and 300 mg, respectively, on day 180. Depending on the dosage, the reductions remained consistent over the entire 12-month study duration [20].

In the recent trial ORION-18, a 12-month double-blind study was conducted across 45 study sites in four countries, targeting adults with LDL levels >70 mg/dl in atherosclerotic cardiovascular patients or LDL >100 mg/dl in high-risk individuals. This placebo-controlled trial was carried out with a single subcutaneous injection of 300 mg inclisiran sodium on days 1, 90, and 270. The trial also reported similar outcomes to previous ORION trials in terms of LDL reduction, with a placebo-adjusted percentage reduction of 57.2% and an absolute change of -60.5 mg/dl from baseline on day 330 of the study. Compared to the placebo, the time-adjusted percentage and absolute reductions from days 90 and 360 were 56.3% and 59.0 mg/dl, respectively. Currently, ORION-13 and ORION-16 are still ongoing in patients of HoFH or HeFH, respectively, with fasting LDL concentrations of >130 mg/dl. The study's goal is to find out how much LDL-C and other markers change over time, as well as the carotid intima-media thickness (cIMT), in teens with HoFH and HeFH using 2D ultrasonography [21].

Pharmacologic Properties of Inclisiran

ORION-14 trial investigated the pharmacokinetics (PK), pharmacodynamics (PD), and safety of single doses of 100 mg and 300 mg of inclisiran sodium in Chinese patients with hypercholesterolemia (LDL levels >100 mg/dl). They performed serial blood sample analysis to evaluate the PK of the drug within 48 hours of dosing. The evaluation revealed that the study subjects' inclisiran concentrations peaked at 8.0 hours and 6.0 hours, respectively, after administering 100 mg and 300 mg of inclisiran sodium. Following the achievement of the maximum concentration, the plasma levels of 100 mg and 300 mg of inclisiran sodium experienced a monoexponential decline, ultimately becoming undetectable at 18.4 hours and 29.6 hours, respectively.

The mean half-life was estimated to be 6.6 and 6.5 hours for 100 mg and 300 mg doses, respectively [19]. In ORION-15, it was found that the concentration in the plasma rises quickly after the drug is injected under the skin. It then drops steadily over time, with a mean plasma half-life of 6.8 (2.0) to 7.6 (0.8) hours [20]. The ORION-1 trial reported that the drug reached its peak plasma levels approximately four hours after dosing and disappeared from plasma within 24-48 hours [12]. However, no other ORION trial has evaluated the pharmacological properties of inclisiran.

Impact of Inclisiran on PCSK9 Reduction

The major secondary endpoint in the ORION trials is the impact of inclisiran on PCSK9, which plays a vital role in the drug's clinical effects. The first trial, ORION-1, assessed the changes in PCSK9 levels as a secondary outcome in patients with elevated LDL levels. The study reported a mean percentage reduction in PCSK 9 levels of 59.6% to 68.7% fourteen days after a single subcutaneous dose of inclisiran, ranging from 100 mg to 500 mg, compared to a 3.8% increase in levels with placebo. By day 30, there was a further increase in reduction, from 66.2% to 74.0% below baseline, with similar reductions observed on days 60 and 90. However, the reduction dropped to between 47.9% and 59.3% by the end of day 180. Subjects who received a second dose of inclisiran sodium showed higher reductions.

These reductions were found to be maintained at greater than 40% by day 240, thereby producing prolonged effects [12]. In the one-year follow-up period of the ORION-1 trial, the percentage reduction in PCSK9 levels ranged from 17.5% to 29.7% and 15.8% to 37.7%, with time-averaged reductions from day 30 to 360 ranging from 44.5% to 55.9% and 43.1% to 60.5% in the single-dose and double-dose groups, respectively, at day 360. According to study results, around 90%-100% of the subjects in the single dose group and 96.6%-100% in the double dose groups have not returned to levels 20% within the baseline at day 180, which dropped to 60.0%-75.0% and 69.5%-86.3% of those receiving single or double dose, respectively [13]. In the four-year extension study ORION-3, the mean percentage reduction in the concentration of PCSK9 was found to be 62.2% to 77.8% over the four years of trial, ensuring sustained results with twice-yearly dosing [14].

In the similar trials ORION-10 and ORION-11, there was a 69.8% (between-group difference of -83.3%) and 63.6% (between-group difference of -79.3%) reduction in PCSK9 levels from baseline at day 510, respectively [15]. In trials ORION-5 and ORION-9 conducted in HoFH and HeFH subjects, respectively, the levels dropped significantly with inclisiran. In the ORION-5 trial, the placebo-corrected absolute change in levels from baseline at day 180 ranged between -304.4 g/L and -390.4 g/L; meanwhile, the percentage change ranged between -60.6% and -92.3% [16]. ORION-9 reported similar outcomes as ORION-5, with a percentage decrease of 60.7%, an absolute decrease of 282.6 g/L, and a time-averaged difference between day 90 and 540 of -284.6 g/L in the inclisiran group, suggesting the role of the drug in patients with HoFH and HeFH [17].

In the ORION-14 and ORION-15 trials, significant reductions in PCSK9 levels were observed following a single dose of subcutaneous injections depending on the dose. In the former trial, the largest reduction of 80.3% was observed in the group receiving a 300 mg dose at day 30. When compared to placebo, statistically significant reductions were observed with a mean difference of 71.6% for 100 mg groups and 81.6% for the 300 mg group at day 30, and the reductions sustained till day 90 with a mean difference from placebo of 60.2% in the 100 mg group and 76.7% in the 300 mg group [19].

The latter trial showed similar results to ORION-14 with a placebo-adjusted percentage change in PCSK9 at day 180 from the baseline of -66.3% (95% CI: -72.6%, -60.1%; P 0.0001), -73.9% (95% CI: -79.3%, -68.4%; P 0.0001), and -79.2% (95% CI: -84.8%, -73.7%; P 0.0001) for doses 100mg, 200mg, and 300mg, respectively, implying a greater reduction with a higher dose of inclisiran. The reductions were observed from day 14, with sustained reduction maintained over approximately 12 months with a dose-response relationship [20]. The recent 12-month double-blinded trial ORION-18, has similar outcomes on the levels of PCSK9. The placebo-adjusted percentage reduction in the levels from baseline at day 330 was 78.3% (P 0.001), with a mean difference in percentage ranging from 66.5% to 83.3% over the study period between the treatment and placebo arms [21].

With the evidence collected above, Inclisiran reduced the levels of PCSK9 levels significantly while maintaining the results achieved over a long duration, and the response achieved is directly proportional to the dose administered.

Other Parameters

Inclisiran was found to not only reduce the levels of LDL-C and PCSK9 but also extend its impact on the levels of apolipoproteinβ, triglycerides, high-density cholesterol (HDL), and other parameters. The results have been reported in various ORION trials. In the ORION-1 trial, significant reductions in non-HDL cholesterol and apo B have been noted, and the same was reported in the four-year follow-up study ORION 3, with mean percentage reductions in non-HDL cholesterol ranging from -41.7% to -30% and apo B ranging from -40.4% to -26.5% throughout the study, accounting for a mean absolute change ranging from -1.72 mmol/L to -1.23 mmol/L and -42.6 mg/dl to -27.9 mmol/dl, respectively [12,14]. The changes were observed early in the study and maintained throughout.

The similar trials ORION-10 and ORION-11 reported lowered levels of triglycerides and lipoprotein (a), while increasing the HDL cholesterol levels by the end of the study with no mention of apolipoprotein B or non-HDL cholesterol levels [15]. Meanwhile, ORION-9 mentioned the association of inclisiran with total cholesterol, non-HDL cholesterol, Apo B, and triglyceride levels, which were reduced with inclisiran with a median lipoprotein (a) reduction of 17.2% [17]. However, the ORION-9 similar trial ORION-5 has declared no statistically significant placebo-corrected absolute or percentage changes in Apo B, non-HDL-C, lipoprotein(a), and total cholesterol at any point in time [16]. It was also noted that the pharmacological trials ORION-14 and ORION 15 have no mention of the above secondary outcomes. The recent trial ORION-18 reported a significant reduction in the levels of total cholesterol by 33.0%, ApoB by 42.3%, and non-HDL cholesterol levels by 9.1%, with the ratio of lipoprotein (a) to baseline being approximately 41% lower in the inclisiran group. (P<0x04>0.001 for all). The mentioned percentage changes were found to be consistent with the percentage changes [21].

The association of C-reactive protein was mentioned in the trials ORION-1 and ORION-9, where no statistically significant association was able to be established. 

Adverse Effects of Inclisiran

A newly released drug has to undergo various safety trials while estimating the possible treatment-related events in the patients. Also, with increased dosages of drugs to improve efficacy, the probability of incidence and severity of adverse events rises. Hence the ORION trials have addressed the issue of safety recording the events that occurred during the trials. In the ORION-1 trial, the adverse events noted in the inclisiran group were similar to the placebo group (76% in each group, with a higher number of serious events in the inclisiran group (11% vs. 8% in the inclisiran vs. placebo groups). The most common events noted were myalgia, diarrhea, headache, back pain, nasopharyngitis, hypertension, and dizziness, with no significant difference in the incidence between the two groups.

However, the incidence of injection-related adverse events was higher in the inclisiran and increased with the number of doses (4% in patients receiving a single dose vs. 7% in those receiving a double dose) as compared to no reactions in the placebo group. Interestingly, a transient rise in the levels of hepatic aspartate aminotransferase in four subjects to three times the upper limit of the normal range was noted (one patient from the placebo arm with elevated aspartate aminotransferase and three patients from the treatment arm with elevations in both alanine and aspartate transaminases) with no increase in bilirubin levels of both arms [12]. The one-year follow-up of the above trial revealed a similar incidence of adverse events and remained stable during follow-up to day 360. However, the four-year follow-up revealed adverse events in 97% (275 out of 284) of the patients in the treatment group and 92% (80 out of 87) of the patients who were switched from the placebo to the inclisiran arm.

The majority of events reported were found to be single cases, with mild to moderate severity, and self-limited. The most common event was noted to be nasopharyngitis in 19% of the patients (55 out of 284) in the inclisiran-only arm and hypertension in 20% of the patients in the switching arm (17 out of 87). The events that could be possibly related to the study medication as determined by the investigator occurred in 28% (79 out of 284 patients) in the Inclisiran am and 25% (22 out of 87 patients) in the switching arm, where most of them were general disorders or injection site reactions such as pain, reaction, and erythema. In the entire four-year duration, the proportion of patients with at least one treatment-emergent adverse event at the injection site was 14% (39 out of 284) in the inclisiran arm, 8% during the first year on placebo, and 14% in the following three years on inclisiran in the switching arm. The incidence of serious treatment-emergent adverse events increased from 11% in ORION-1 to 37% (104 out of 284) in the inclisiran arm and from 8% to 34% (30 out of 87) in the switching arm. A total of eight deaths were reported, and none of them were found to be study-related.

However, in the serious treatment-emergent study, drug-related events occurred only in 3% of patients in the inclisiran arm and 1% of patients in the switching arm. The patients include one with accessory pathway-mediated tachycardia considered to be exacerbated by the study medication, one with acute cholecystitis in a known case of gallstones, one case of hepatic fibrosis in a patient with fatty liver disease, and one case of increased liver enzymes, alanine aminotransferase (ALT) and aspartate aminotransferase (AST), greater than 5x the upper limit in a patient with hepatitis C combined with heavy alcohol intake. At least one treatment-emergent hepatic event was reported in 10% (28 out of 284 patients) in the inclisiran arm and 9% (eight out of 87 patients in the switching arm), with most of the events being mild to moderate elevations in liver enzymes [14].

The later trials ORION-10 and ORION-11 reported similar adverse events in the inclisiran and placebo groups, although more injection site events have been reported in the inclisiran arm as compared to the placebo (2.6% vs. 0.9% in ORION-10 and 4.7% vs. 0.5% in ORION-11). The reactions were noted to be mild and severe or persistent. Around 73.5% (574 out of 781) individuals receiving inclisiran and 74.85 (582 out of 778) reported at least one event during the ORION-10 trial, and 82.7% (671 out of 811) receiving inclisiran and 81.5% (655 out of 804) receiving placebo in ORION-11. Serious adverse events were reported in 22.4% (175 patients) receiving inclisiran, 26.3% (205 patients) receiving a placebo in ORION-10, and 22.3% (181 patients) and 22.5% (181 patients) in the placebo group of ORION-11 [15].

Also, in the trials ORION-5 and ORION-9 in the HoFH and HeFH populations, it is reported that no significant difference in the incidence of adverse events has been recorded between treatment and placebo groups throughout the entire study, with three reported deaths in part two of the ORION-5 trial, and none of them were found to be related to inclisiran treatment, attributed to other factors such as multiple organ dysfunction syndrome, sudden cardiac death, and viral pneumonia. The most common treatment-emergent adverse events in part one of ORION-5 were viral respiratory tract infection (n=2, 54% in the inclisiran arm; n=2, 10.5% in the placebo arm), pyrexia (n=2, 5.4% in the inclisiran arm; 0% in the placebo), and in the part-two phase, coronavirus infection was noted in three patients (8.8%) in the inclisiran arm and one patient (5.3%) in the placebo-inclisiran arm. An increase in International Normalized Ratio (INR) was seen in two patients (5.9%), and one patient (5.3%) in the inclisiran-only and placebo-inclisiran arms, respectively. Injection site adverse events (erythema) were noted in only one patient (2.9%) of the inclisiran-only arm throughout the entire study [16].

Meanwhile, in the later trial ORION-9, 76.8% (185 out of 241) patients in the treatment arm and 71.7% (172 out of 240) patients in the placebo arm reported adverse events during the trial. The majority of the events (94.6% in inclisiran and 91.9% in placebo groups) were either mild or moderate. Surprisingly, the number of serious adverse events was lower in the placebo groups compared to the treatment arm (13.8% vs. 7.5%), which included one death in each group, neither of which was proven to be related to the trial intervention. However, a huge difference in the incidence of injection site adverse events between the treatment and placebo arms (17.0% vs. 1.7%, with a between-group difference of 15.3%) was noted, with the majority (90.2%) being mild and severe or persistent [17]. In the combined follow-up of ORION-3, ORION-9, ORION-10, and ORION-11, termed ORION-8, approximately 77.8% (n=2548) had treatment-emergent adverse events, 30.2% (n=989) had treatment-emergent serious adverse events, 5.0% (n=165) had fatal TEAEs, and 2.4% (n=80) had TEAEs that led to discontinuation of study medication.

The most common TEAEs were COVID-19 infection (13.8%), inadequate diabetes mellitus control (7%), and hypertension (7%), and the frequent serious treatment-emergent adverse events were coronary artery disease (2%), COVID-19 (1.5%), and acute myocardial infarction (1.3%). During the study, the following safety topics of interest with the use of trial medication were identified: injection site events (5.9%), hepatic events (5.1%), and worsening or de novo diabetes mellitus (17.8%). When adjusted to exposure rate per 100 patient years for > one drug-related TEAE, the rate of incidence of TEAEs is as follows: TEAE [5.1 (3.4-7.5) vs. 4.8 (4.4-5.3)], ≥ 1 serious TEAE [12.8 (9.8-16.4) vs. 12.4 (11.6-13.2)], and 1 TEAE leading to study treatment discontinuation [0.7 (0.2-1.7) vs. 0.7 (0.5-0.8)] were comparable among patients who were ADA-positive (n=162) and ADA-negative (n=2783) [18].

The trials conducted in the Chinese and Japanese populations (ORION-14 and ORION-15, respectively) revealed similar rates of incidence in both treatment and placebo arms. In ORION-14, the incidence of adverse events was 66.7% and 66.0% in the treatment and placebo arms, respectively, with no serious events or treatment discontinuation. Two patients (13.3%) in the 100 mg inclisiran arm and one patient (10.0%) in the placebo arm experienced a transient elevation in blood uric acid levels, and in the 300 mg inclisiran group, anemia, and abnormal hepatic function were experienced by two people each. The elevated liver enzymes were found to be mild, asymptomatic, and transient (returned to baseline within 30 days). Also, two injection site events were reported (one from each group-100 mg inclisiran and placebo) [19]. And the later trial, ORION-15, reported the incidence of adverse events as: 89.1% (n=49) in the 100 mg inclisiran group, 83.2% (n=84) in the 200 mg inclisiran group, 80.8% (n=80) in the 300 mg inclisiran group, and 84.2% (n=48) in the placebo group.

Severe events were reported in only three patients with inclisiran (one patient from the 200 mg inclisiran group experienced rhabdomyolysis, circulatory collapse, and peripheral artery occlusion, and one patient from the 300 mg inclisiran group reported unstable angina, congestive cardiac failure, infection, and electrolyte imbalance; the third patient reported acute pancreatitis), and four patients in placebo, which are considered irrelevant to study medication. The most common events reported at a dose of inclisiran sodium were pyrexia (18.2%, 11.9%, 14.5%, 12.3%), inadequate diabetes mellitus control (15.2%, 10.9%, 12.7%, 17.5%), and diabetes mellitus (12.1%, 11.9%, 20.0%, 8.8%) (the reported rates of incidence were of 300mg, 200mg, 100mg inclisiran sodium, placebo respectively). Though unrelated to treatment medication, two patients discontinued due to treatment-emergent adverse events (one patient with chronic gastritis in the 200 mg treatment arm, and one patient with idiopathic urticaria in the 300 mg treatment arm). The incidence of the most common event (injection site reactions) was as follows: inclisiran sodium 100 mg: 5.5% (n=3), inclisiran sodium 200 mg: 10.9% (n=11), inclisiran sodium 300 mg: 5.1% (n=5), and placebo: 3.5% (n=2).

The reactions were mild and transient. A total of 27 serious events were reported in 22 patients, and none of the events were considered treatment-related with one death of unknown cause. It was noted that TEAEs associated with hepatic events were higher in the 300 mg inclisiran group. Three patients reported three hepatic events, and two of them had steatosis at baseline. And no correlation has been found between the rate of incidence of TEAE and the dose of inclisiran [20]. In the recent trial ORION-18, over a study period of one year, 73.5% of patients in the inclisiran group and 66.7% in the placebo group have reported mild to moderate TEAEs, with only 4.7% (n=8) and 2.3% (n=4) in the inclisiran and placebo groups reporting serious events.

The most common events in both arms were diabetes mellitus, increased blood creatinine phosphokinase, inadequate control of diabetes mellitus, upper respiratory tract infection, urinary tract infection, and insomnia. As determined by the investigator, TEAEs related to the study drug were reported in 11.8% and 10.3% of treatment and placebo arms, respectively. However, in the inclisiran group, the most common drug-related TEAEs were injection site events (pain), elevated liver enzymes by <3x the upper limit of normal, and increased body weight. The Inclisiran group reported higher rates of injection-related adverse events when compared to placebo (2.9% vs. 0.6%) and were mild and transient. The most common event in the placebo group was elevated blood creatinine phosphokinase (2.3%). Serious TEAEs were reported in 16.5% and 9.8% of the inclisiran and placebo arms, respectively [21].

Formation of autoantibodies against the RNAi molecule inclisiran was another vital adverse event to be considered, as that would render the treatment ineffective in those who developed antibodies to the drug. However, it can be seen that only one patient among the subjects of ORION-1 tested positive for antibodies before the first dose of inclisiran, and no other case of positive antibodies was reported. ORION-10 and ORION-11 trials reported an incidence of 2.0% and 2.5%, respectively. The frequency was found to be similar in pre- and post-treatment samples. In the post-treatment samples, antibody titer was found to be low. Often transient, not associated with changes in any pharmacologic or clinical variables. There were no treatment-boosted antidrug antibodies noted. However, ORION-9 has reported transiently low titers of antidrug antibodies in 2.6% of the samples (25 samples from 18 patients), but they were not considered to be due to treatment with inclisiran. And no antibodies were detected in any of the samples in the similar trial, ORION-5.

The cumulative follow-up trial of the above studies excluding ORION-5 (ORION-8) revealed an incidence of 5.5% (162/2945), with 3.8% of patients exhibiting a transient response and 1.7% exhibiting a persistent response. Also, it was determined that the levels of anti-drug antibodies have not affected the primary outcome, mean percentage change, and mean absolute change in the levels of LDL-C from baseline (percentage change in levels of LDL-C was -47.4% (-52.2 to -42.6), -49.4% (-50.6 to -48.2), and mean absolute change in levels of LDL-C from baseline were -1.38 mmol/L (-1.55 to -1.22 mmol/L), -1.44 mmol/L (-1.48 to -1.40 mmol/L) in ADA positive (n=138) and ADA negative (n=2307) individuals, respectively. No autoantibodies were detected in the ORION-14 trial. However, in the ORION-15 trial, five out of 255 patients (three from the 200 mg inclisiran group and two from the 300 mg group) had confirmed preexisting antidrug antibodies with no correlation between positive status and TEAEs.

Limitations of the study**

The study only included randomized control trials where the drug was originally administered to the subjects with follow-up to evaluate safety and efficacy. However, this study did not analyze the pharmacological properties of the drug and its interaction with other medications. Also, this study did not consider existing comorbidities (other than ASCVD) and their impact on the study drug, cost-effectiveness, and drug compliance due to infrequent dosing, which need to be addressed. And few of the trials were conducted by the same group of individuals which can add to potential bias in the reported outcomes. However, this study provided detailed insight into the findings of the clinical trials and included a complete summary of the original ORION trials together.

## Conclusions

After carefully examining the findings of clinical trials presented above, it is safe to conclude that inclisiran has a strong potential in the treatment of hypercholesterolemia with twice-yearly dosing, improving the compliance of pharmacological treatment and providing higher efficacy with a similar safety profile as compared to placebo or other conventional therapies. Also, with higher doses, greater reductions in levels of LDL-C and other parameters could be attained without any significant difference in the incidence of adverse events. However, longer follow-up studies are still needed to evaluate long-term efficacy and safety, especially given potential issues of elevated liver enzymes, the formation of anti-drug antibodies with long-term use, and other unknown possible treatment-emergent adverse events.
